# The role of microRNAs in intrahepatic cholangiocarcinoma

**DOI:** 10.1111/jcmm.12951

**Published:** 2016-09-13

**Authors:** Zheng Li, Jianxiong Shen, Matthew T.V. Chan, William Ka Kei Wu

**Affiliations:** ^1^ Department of Orthopedics SurgeryPeking Union Medical College HospitalChinese Academy of Medical Sciences and Peking Union Medical CollegeBeijingChina; ^2^ Department of Anaesthesia and Intensive CareThe Chinese University of Hong KongHong KongChina; ^3^ State Key Laboratory of Digestive DaaseLKS Institute of Health SciencesThe Chinese University of Hong KongHong KongChina

**Keywords:** Intrahepatic cholangiocarcinoma, microRNAs, proliferation, apoptosis, metastasis, prognosis

## Abstract

Intrahepatic cholangiocarcinoma (ICC) is the second most common primary hepatic malignancy with poor prognosis. Despite improvements in its diagnosis and therapy, the prognosis for ICC patients remains poor. An improved understanding of ICC pathogenesis and consequential identification of novel therapeutic targets would improve the prognosis of ICC patients. MicroRNAs (miRNAs) are a class of highly conserved, endogenous, small non‐coding RNA molecules of 18–23 nucleotides in length, which regulate gene expression through complementary base‐pairing with target messenger RNAs and subsequent gene silencing. Several studies have shown deregulated expression of miRNAs in ICC cell lines and tissues, in which these miRNAs play important roles in ICC apoptosis, cell proliferation, invasion, migration and metastasis. In this review, we illustrate the potential role of miRNA in the pathogenesis of ICC and explore the possibilities of using miRNAs as prognostic and diagnostic markers, as well as therapeutic targets in ICC.

## Introduction

Intrahepatic cholangiocarcinoma (ICC) originating from cholangiocytes is the second most common primary tumour of the liver 1, 2, 3, 4. It comprises approximately 5–10% of liver cancers, and both its worldwide incidence and mortality rate have been increasing over the past three decades 5, 6, 7. Surgical resection is still considered to be the only potential curative treatment. However, the prognosis of ICC patients is poor with 5‐year survival of only 25–35% in most studies 8, 9, 10. As there is currently no molecular marker for its early diagnosis, elucidating the molecular pathogenesis of ICC may be crucial for identifying new molecular markers for early diagnosis and ultimately improving the prognosis of ICC patients 11, 12, 13, 14, 15.

MicroRNAs (miRNAs) belong to a new class of small non‐coding, endogenous RNAs comprising 18–23 nucleotides that negatively regulate gene expression through inducing mRNA degradation or repressing translation by annealing with the complementary sites in 3′‐untranslated regions (3′‐UTRs) of target mRNAs 16, 17, 18, 19. Increasing evidence has suggested that miRNAs play crucial roles in many biological processes, such as development, cell proliferation, invasion, migration and differentiation 20, 21, 22, 23, 24. Mutations of miRNA‐encoding genes or aberrant expression of miRNAs have been well described in various tumours, including gastric, breast and lung cancers, as well as glioblastoma and melanoma 25, 26, 27. However, the expression and functional roles of miRNAs in the development of ICC remain elusive. In this review, we focus on recent studies related to miRNAs involved in ICC development and discuss the potential use of miRNAs as prognostic biomarkers and treatment strategies for ICC.

## Biogenesis of miRNA

Previous data showed that miRNA synthesis and maturation consists of a stepwise process that is compartmentalized between the nucleus and the cytoplasm 28, 29, 30. The pri‐miRNA (primary miRNA transcript) is transcribed by RNA polymerase II in the nucleus, resulting in a transcript with a stem‐loop structure of about 70 nucleotides, a 5′ 7‐methylguanylate cap and a 3′‐polyadenylated tail 30, 31, 32. A ribonucleoprotein RNase III enzyme, Drosha, and its cofactor DiGeorge syndrome critical region 8 (DGCR8) mediate the release of the stem‐loop intermediate known as the pre‐miRNA (precursor miRNA) 33, 34, 35. The pre‐miRNA is then shuttled from the nucleus to the cytoplasm *via* exportin‐5 36, 37, 38. The terminal loop is cleaved from the pre‐miRNA by Dicer (another ribonucleoprotein RNase III enzyme) in the cytoplasm 39, 40, 41.

## miRNAs in intrahepatic cholangiocarcinoma

Deregulated miRNAs in ICC were listed in Table 1. The first report on miRNA expression profiling in human ICC was performed by Chen *et al*. using 27 ICC tissues, 10 normal cholangiocyte samples and 8 normal liver tissues 42. miRNA profiling revealed 18 and 20 significantly up‐regulated and down‐regulated miRNAs, respectively, in ICC tissues compared with the normal samples. Furthermore, they compared the miRNA expression between cholangiocytes and normal liver tissues to identify some tissue‐specific miRNAs and revealed 21 miRNAs differentially expressed between these two tissue types.

**Table 1:** MiRNA expression profiles in intrahepatic cholangiocarcinoma (ICC)

Num	Method	Sample	Up‐regulated	Down‐regulated	Reference
1	Microarray	Primary ICC	mir‐21 mir‐142‐3p mir‐25 mir‐15a mir‐193 mir‐17‐5p mir‐374 mir‐106a mir‐224 mir‐130b mir‐19a mir‐331 mir‐324‐5p mir‐20 mir‐17‐3p mir‐223 mir‐15b mir‐103	mir‐98 mir‐204 mir‐338 mir‐198 mir‐302d mir‐328 mir‐337 mir‐302b mir‐184 mir‐320 mir‐371 mir‐185mir‐222 mir‐214 mir‐373 mir‐145 miR‐200c let‐7a let‐7b mir‐197	42
2	Microarray	ICC cell lines (HuCCT1 and MEC)	miR‐22 miR‐125a miR‐127 miR‐199a miR‐214 miR‐376a miR‐199a* miR‐424		43
3	Microarray	ICC tissues	miR‐660 miR‐425 miR‐93 miR‐494	miR‐150 miR‐638 miR‐4459 miR‐4530 miR‐378c	46
4	Microarray	ICC tissues	miR‐566 miR‐423‐5p miR‐612 miR‐765 miR‐625‐3p miR‐491‐5p miR‐188‐5p miR‐92b‐5p miR‐675‐5p miR‐331‐3p	miR‐141‐3p miR‐497‐5p miR‐29a‐3p let‐7a‐5p miR‐19b‐3p miR‐103a‐3p miR‐130a‐3p let‐7d‐5p miR‐100‐5p miR‐26b‐5p let‐7e‐5p miR‐24‐3p miR‐101‐3p let‐7f‐5p miR‐99a‐5p miR‐338‐3p miR‐29c‐3p miR‐26a‐5p miR‐451a miR‐143‐3p	47
5	Microarray	ICC tissues	miR‐141‐3p miR‐141‐5p miR‐200c‐3p miR‐577 miR‐200b‐5p miR‐200a‐5p miR‐135b‐5p miR‐429 miR‐200a‐3p miR‐196a‐1‐5p	miR‐483‐5p miR‐122‐5p miR‐548aq‐3p miR‐422 miR‐483‐3p miR‐675‐3p miR‐383 miR‐548aq‐3p miR‐885‐5p miR‐1275	48

Further study on miRNAs expression profiling identified a number of significantly deregulated miRNAs in two ICC cell lines (HuCCT1 and MEC) as compared with a normal intrahepatic biliary epithelial cell line (HIBEpiC) 43. The investigators found 27 miRNAs that were expressed exclusively or predominantly in each cell line, and miR‐22, miR‐125a, miR‐127, miR‐199a, miR‐199a*, miR‐214, miR‐376 and miR‐424 were down‐regulated in the ICC cell lines compared with HIBEpiC using real‐time PCR.

Oishi *et al*. 44 used the NanoStringn Counter microRNA expression assay platform to examine gene expression profiles of 23 ICC samples with validation in an independent cohort of 68 ICC cases. Unsupervised clustering analysis based on the expression of all 700 human mature miRNAs revealed that ICCs could be divided into two main clusters, namely hepatic stem cell‐like ICC and mature hepatocyte‐like ICC, which could be differentiated by 23 miRNAs.

Plieskatt *et al*. 45 studied miRNA expression profiling in relation to histologic grades and subtypes of ICC (well‐differentiated, moderately differentiated and papillary ICC) by microarray. They found that each histologic grade and subtype of ICC displayed a distinct miRNA profile, without common deregulated miRNA. Moderately differentiated ICC showed the greatest miRNA deregulation in quantity and magnitude, followed by the papillary subtype, and then the well‐differentiated ICC. Moreover, when ICC tumour tissues were compared with adjacent non‐tumour tissue, similar miRNA dysregulation profiles were observed.

Wang *et al*. 46 profiled miRNA expression in three pairs of ICC tissues and peritumoral normal tissues using the microarray platform. They found 10 deregulated miRNAs in the ICC tissues, in which the most significantly down‐regulated miRNAs were miR‐150, miR‐638, miR‐4459, miR‐4530 and miR‐378c, while the overexpressed miRNAs involved miR‐660, miR‐425, miR‐93 and miR‐494.

Karakatsanis *et al*. 47 demonstrated that miR‐21, miR‐31 and miR‐223 were overexpressed in 21 patients with primary ICC, whereas miR‐122, miR‐145, miR‐200c, miR‐221 and miR‐222 were down‐regulated. However, miR‐21, miR‐31 and miR‐223 did not show correlation with clinicopathological features.

Recently, Zhang *et al*. 48 conducted miRNA expression profiling in 63 human ICCs and nine normal intrahepatic bile duct samples (NIBD) using a custom microarray containing 1094 probes. Expression analysis showed 158 differentially expressed miRNAs between ICC and NIBD, with 77 up‐regulated and 81 down‐regulated miRNAs. From the 158 differentially expressed miRNAs, a 30‐miRNA signature consisted of 10 up‐regulated and 20 down‐regulated miRNAs in ICC was established for distinguishing ICC from NIBD with 100% accuracy. A separate 3‐miRNA signature was identified for predicting prognosis in ICC. Based on the 3‐miRNA signature, a formula was constructed to compute a risk score for each patient. Patients with high‐risk scores had significantly lower overall survival and disease‐free survival than those with low risk scores. Moreover, they showed that three miRNAs (miR‐675‐5p, miR‐652‐3p and miR‐338‐3p) were significantly associated with overall survival. Of the three miRNAs, miR‐675‐5p was up‐regulated and negatively associated with overall survival, while the other two (miR‐652‐3p and miR‐338‐3p) were down‐regulated and positively associated with overall survival.

Plieskatt *et al*. 49 comprehensively profiled miRNA expression in ICC tumour tissues using small RNA sequencing and validated the expression profiles using quantitative PCR on matched plasma samples. Distinct miRNA profiles were associated with increasing histological differentiation of ICC. They observed that histologically normal tissues adjacent to ICC tumours displayed miRNA expression profiles more similar to tumours than liver tissues from healthy donors. In plasma samples, an 8‐miRNA signature was associated with ICC, regardless of the degree of histological differentiation of its matched tissue, forming the basis of a circulating miRNA‐based biomarker for ICC.

A number of aberrantly expressed miRNAs have been reported to function as tumour suppressors or oncogenes in ICC (Table 2) *via* derepressing or suppressing important signalling mediators along specific signalling pathways pertinent to cancer development.

**Table 2:** Functional characterization of the deregulated miRNAs in intrahepatic cholangiocarcinoma (ICC)

Name	Up‐ or down‐regulation	Target gene	Role	Reference
miR‐675‐5p	Up		Oncogene	47
miR‐652‐3p	Down		Tumour suppressor	47
miR‐338‐3p	Down		Tumour suppressor	47
miR‐31	Up	RASA1	Oncogene	49
miR‐150	Up		Oncogene	46
miR‐21	Up	PTPN14, PTEN	Oncogene	50
miR‐204	Down	Mcl‐1, Bcl‐2	Tumour suppressor	42
miR‐320	Down	Mcl‐1, Bcl‐2	Tumour suppressor	42
miR‐21	Up		Oncogene	51
miR‐31, miR‐223	Up		Oncogene	51
miR‐122, miR‐145, miR‐200c, miR‐221, miR‐222	Down		Tumour suppressor	51
miR‐214	Down	Twist	Tumour suppressor	52
miR‐200c	Down	NCAM1	Tumour suppressor	44
miR‐124	Down	SMYD3	Tumour suppressor	53
miR‐376c	Down	GRB2	Tumour suppressor	54
miR‐204	Down	slug	Tumour suppressor	55
miR‐605	Down	PSMD10	Tumour suppressor	56

## Up‐regulated miRNAs in intrahepatic cholangiocarcinoma

Hu *et al*. 50 reported that the expression of miR‐31 was significantly up‐regulated in ICC tissues and the human ICC cell line HCCC‐9810, when compared with normal adjacent tissues. Down‐regulation of miR‐31 significantly inhibited cell proliferation and promoted apoptosis in HCCC‐9810 cells. RAS p21 GTPase activating protein 1 (RASA1) was identified as a direct target of miR‐31, and there was an inverse correlation between miR‐31 and RASA1 expression during ICC development.

Wang *et al*. 46 showed that miR‐150 levels were significantly higher in the ICC plasma samples as compared with the matched control plasma samples. The diagnostic value of plasma miR‐150 was also analysed. For differentiating ICC from age‐ and gender‐matched normal controls, receiver operator curve (ROC) analysis of plasma miR‐150 revealed the area under the curve (AUC) of 0.764 with a sensitivity of 80.6% and a specificity of 58.1%.

Wang *et al*. 51 showed that miR‐21 plays an important role in the regulation of cell proliferation and tumour growth in ICC, in which miR‐21 could also serve as a diagnostic and prognostic marker as well as a potential therapeutic target. They found that miR‐21 levels were significantly higher in serum of ICC patients. Inhibition of miR‐21 suppressed ICC cell proliferation, induced cell cycle arrest and apoptosis *in vitro* and impaired tumour growth *in vivo*. Furthermore, PTPN14 and PTEN were identified as direct and functional targets of miR‐21. High expression levels of miR‐21 were closely related to adverse clinical features, diminished survival and poor prognosis in ICC patients.

## Down‐regulated miRNAs in intrahepatic cholangiocarcinoma

The biological functions and/or prognostic significance of two down‐regulated miRNAs, namely miR‐204 and miR‐320, have been studied in details 42. Restored expression of miR‐320 and miR‐204 negatively regulated Mcl‐1 and Bcl‐2 expression, respectively, and facilitated chemotherapeutics‐triggered apoptosis.

Li *et al*. 52 found that miR‐214 levels were significantly lower in ICC tissues compared with normal tissues. Moreover, miR‐214 expression was remarkably decreased in primary tumours that subsequently metastasized compared with non‐metastatic ICC. Inhibition of miR‐214 promoted metastasis of human ICC cells and increased the transcript levels of the epithelial–mesenchymal transition‐associated gene *Twist*, and decreased E‐cadherin levels by directly targeting the *Twist* gene. Oishi *et al*. 44 also found that the expression level of miR‐200c was lower in ICC and was associated with overall survival and disease‐free survival in ICC cases. Transient transfection of miR‐200c oligos into HuH28 cells induced reversal of epithelial‐mesenchymal transition (EMT) from a mesenchymal‐like to a cobblestone‐like morphology with suppression of genes that mediate EMT. Functionally, miR‐200c overexpression suppressed cell migration and invasion. Moreover, NCAM1 was identified as a direct target of miR‐200c.

Hepatitis C virus core protein (HCVc) plays an important role in the development of ICC. Zeng *et al*. 53 found that miR‐124 was down‐regulated in HCV‐ICC and the induction of DNMT1 by HCVc mediated the suppression of miR‐124. Ectopic expression of miR‐124 suppressed cell migration and invasion *in vitro* and reduced the protein levels of SMYD3 and downstream target genes (c‐Myc and matrix metallopeptidase 9). Knockdown of SMYD3 inhibited cell migration and invasion resembling that of miR‐124 overexpression.

Iwaki *et al*. 54 demonstrated that miR‐376c was down‐regulated in ICC cell line (HuCCT1) compared with normal intrahepatic biliary epithelial cells (HIBEpiC). Growth factor receptor‐bound protein 2 (GRB2) was identified as a direct target of miR‐376c. miR‐376c overexpression reduced epidermal growth factor (EGF)‐dependent cell migration in HuCCT1 cells. Interleukin 1β and matrix metallopeptidase 9 were possible participants in EGF‐dependent migration of HuCCT1 cells as determined by microarray and subsequent pathway analysis. Bisulfite sequencing showed higher methylation levels of CpG sites upstream of the miR‐376c‐encoding gene in HuCCT1 relative to HIBEpiC cells. Combined treatment with the DNA‐demethylating agent 5‐aza‐2′‐deoxycytidine and the histone deacetylase inhibitor trichostatin A significantly up‐regulated the expression of miR‐376c in HuCCT1 cells.

Qiu *et al*. 55 reported that the expression of miR‐204 was frequently down‐regulated in ICC tissues and the low‐level expression of miR‐204 was significantly associated with lymph node metastasis. miR‐204 overexpression suppressed ICC cell migration and invasion, as well as EMT by regulating slug expression.

Li *et al*. 56 showed that the expression of miR‐605 was expressed at low levels and inversely correlated with the expression of proteasome 26S subunit non‐ATPase 10 (PSMD10) in ICC. Overexpression of miR‐605 inhibited ICC cell proliferation and invasion by regulating PSMD10 expression. Restoration of PSMD10 reversed the phenotypic alteration caused by miR‐605 in ICC cells.

## Serum miRNAs in intrahepatic cholangiocarcinoma

Bernuzzi *et al*.^57^ performed miRNA expression profiling in 90 serum samples [30 primary sclerosing cholangitis (PSC), 30 cholangiocarcinoma and 30 control cases] to found disease‐associated miRNAs (discovery phase). They found that 33 in cholangiocarcinoma, 21 miRNAs differentially expressed in PSC and 26 in both in comparison to control cases and 24 miRNAs differentially expressed between cholangiocarcinoma and PSC. Furthermore, they demonstrated that miR‐194 and miR‐483‐5p showed deregulated expression in cholangiocarcinoma compared with controls.

## Concluding remarks and future perspectives

**Figure 1:**
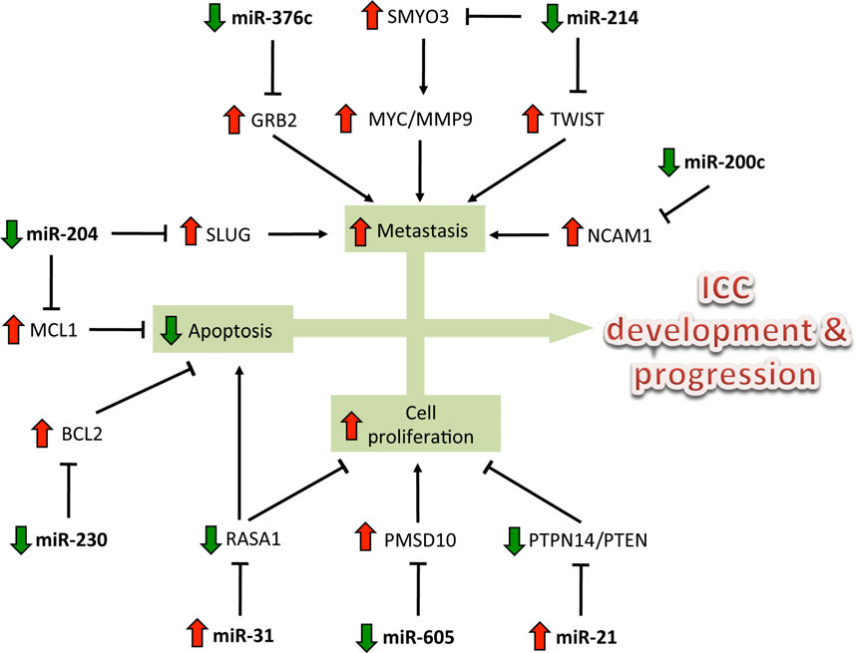
Regulation of tumour cell proliferation, apoptosis and metastasis by miRNAs in the development and progression of intrahepatic cholangiocarcinoma (ICC). miR‐21 and miR‐31 were up‐regulated in the ICC and promote the ICC cell proliferation and invasion and repress the ICC cell apoptosis; miR‐376c, miR‐214, miR‐204, miR‐200c, miR‐230 and miR‐605 were down‐regulated in the ICC and inhibit the ICC cell proliferation and invasion and promote the ICC cell apoptosis.

The dismal prognosis and aggressive progression associated with ICC have led researchers and clinicians to explore new avenues of potential treatment for ICC patients 58, 59, 60. Increasing evidence demonstrated that miRNAs are involved in important biological processes, including cell proliferation, differentiation, migration, invasion and apoptosis 61, 62, 63, 64, 65. As illustrated in Figure 1, altered expression of miRNAs has significant effects on intracellular signalling network and thereby promoting malignant phenotypes in the development and progression of ICC. However, we are still facing many difficulties in miRNA research. In particular, miRNA‐based therapy is not currently available in clinic settings. Nevertheless, with more research efforts to put forth the development of miRNA‐based therapeutics and delivery system, it is hopeful that miRNAs may be used to target specific traits of ICC.

## Conflict of interest

The authors declare no conflict of interest.
